# Correlation of motor-auditory cross-modal and auditory unimodal N1 and mismatch responses of schizophrenic patients and normal subjects: an MEG study

**DOI:** 10.3389/fpsyt.2023.1217307

**Published:** 2023-10-11

**Authors:** Mitsutoshi Okazaki, Masato Yumoto, Yuu Kaneko, Kazushi Maruo

**Affiliations:** ^1^Department of Psychiatry, National Center Hospital of Neurology and Psychiatry, Kodaira, Japan; ^2^Department of Psychiatry, Ome Municipal General Hospital, Ome, Japan; ^3^Department of Clinical Engineering, Faculty of Medical Science and Technology, Gunma Paz University, Takasaki, Japan; ^4^Department of Neurosurgery, National Center Hospital of Neurology and Psychiatry, Kodaira, Japan; ^5^Department of Biostatistics, Faculty of Medicine, University of Tsukuba, Tsukuba, Japan

**Keywords:** schizophrenia, N1, mismatch negativity, magnetoencephalography (MEG), cross-modal context, forward model

## Abstract

**Introduction:**

It has been suggested that the positive symptoms of schizophrenic patients (hallucinations, delusions, and passivity experience) are caused by dysfunction of their internal and external sensory prediction errors. This is often discussed as related to dysfunction of the forward model that executes self-monitoring. Several reports have suggested that dysfunction of the forward model in schizophrenia causes misattributions of self-generated thoughts and actions to external sources. There is some evidence that the forward model can be measured using the electroencephalography (EEG) and magnetoencephalography (MEG) components such as N1 (m) and mismatch negativity (MMN) (m). The objective in this MEG study is to investigate differences in the N1m and MMNm-like activity generated in motor-auditory cross-modal tasks in normal control (NC) subjects and schizophrenic (SC) patients, and compared that activity with N1m and MMNm in the auditory unimodal task.

**Methods:**

The N1m and MMNm/MMNm-like activity were recorded in 15 SC patients and 12 matched NC subjects. The N1m-attenuation effects and peak amplitude of MMNm/MMNm-like activity of the NC and SC groups were compared. Additionally, correlations between MEG measures (N1m suppression rate, MMNm, and MMNm-like activity) and clinical variables (Positive and Negative Syndrome Scale (PANSS) scores and antipsychotic drug (APD) dosages) in SC patients were investigated.

**Results:**

It was found that (i) there was no significant difference in N1m-attenuation for the NC and SC groups, and that (ii) MMNm in the unimodal task in the SC group was significantly smaller than that in the NC group. Further, the MMNm-like activity in the cross-modal task was smaller than that of the MMNm in the unimodal task in the NC group, but there was no significant difference in the SC group. The PANSS positive symptoms and general psychopathology score were moderately negatively correlated with the amplitudes of the MMNm-like activity, and the APD dosage was moderately negatively correlated with the N1m suppression rate. However, none of these correlations reached statistical significance.

**Discussion:**

The findings suggest that schizophrenic patients perform altered predictive processes differently from healthy subjects in latencies reflecting MMNm, depending on whether they are under forward model generation or not. This may support the hypothesis that schizophrenic patients tend to misattribute their inner experience to external agents, thus leading to the characteristic schizophrenia symptoms.

## Introduction

Most schizophrenic patients experience hallucinations, delusions, and passivity experiences as prominent symptoms (so-called positive symptoms) (1, 2). They often interpret apparently inexplicable experiences they have as “alien thoughts inserted into their mind” (3). These psychopathological descriptions have led to the idea that these symptoms come from patient interpretations of internally generated voices or thoughts as external voices and that their movements and speech as externally caused (1, 2). Frith (3) suggested that this misinterpretation is due to a failure of the self-monitoring system in schizophrenic patients who cannot distinguish between external events and perceptual changes caused by their own actions.

The execution of self-monitoring is often explained with a “forward model” system, in which an efference copy of a motor command is used to predict upcoming sensory consequences of self-initiated motor acts (corollary discharge). Several studies have suggested a dysfunction of the forward model in schizophrenic patients (1, 2, 4–10). Event-related potential studies in normal subjects found that the amplitudes of auditory N1 and its magnetoencephalographic (MEG) equivalent N1m component for self-initiated sounds were significantly attenuated compared with that for externally initiated sounds. These findings have been discussed in relation to the internal forward model mechanisms (11–13). Ford et al. (7) found that reduced N1 suppression to tones delivered by button pressing compared with tones played back was smaller in schizophrenic patients than in normal controls. They suggested that the results reflect failure of the forward model in schizophrenic patients. These reports have discussed that the dysfunction of the forward model in schizophrenia causes misattributions of self-generated thoughts and actions to external sources. For example, it has been hypothesized that this auditory dysfunction of the forward model causes schizophrenic patients to misattribute inner speech as external voices (i.e., auditory hallucinations).

However, it is also well-known finding that a reduction in a mismatch negativity (MMN) and its MEG counterpart MMNm, which is an auditory change-detection responses, was smaller in schizophrenic patients than in normal controls (14–17). Some reports have suggested that the reduction of MMN(m) in schizophrenia is related to impairment of prediction errors (18–20). The predictive coding model theory has also been applied to explanations of the psychopathological phenomena of schizophrenia (7, 21, 22). The MMN(m) in schizophrenic patients has also been discussed in relation to their psychiatric symptoms, especially positive symptoms whose main symptoms are hallucinations and delusional experiences (23–26).

The MMN(m) has usually been measured as a pre-attentive, automatic response in a unimodal paradigm. In an MEG study, Yumoto et al. (27) examined whether the deviant occurrences in the motor-auditory cross-modal oddball paradigm could elicit prediction-driven responses. In their experiment, subjects were asked to press either of two buttons based on their choice and pace and to listen to the tone (A or B) following the pressing of the button, with tones of A and B being, respectively, assigned beforehand to a specific button as the standard, within 15% deviance (switching of the tone). They found that normal subjects elicited MMNm-like activity when they performed a motor-auditory mismatch, and interpreted this reaction as a temporal internal model based on the motor-auditory rule and suggested that auditory perception is modulated by top-down prediction processes in motor-auditory contexts.

Randeniya et al. (28) suggested that both reduced N1-attenuation to self-generated sounds and MMN amplitudes in schizophrenic patients are symptomatic of aberrant internal and external sensory prediction errors, which explain their notion of imprecise belief formation (externally encroaching hallucinations and delusions). The N1(m)-attenuation to self-generated sounds and generation of MMN(m) are usually detected by different paradigms and have not been discussed as a series of responses even though they are very close components on the time scale. Differently, in the study of Yumoto et al. (27), it was possible to confirm MMN(m)-like activity under conditions following reduced N1(m)- attenuation to self-generated sounds due to the motion-related forward model. This MMN(m)-like activity, either by itself or compared to conventional unimodal MMN(m), may add new insights in the diagnosis or symptom assessment of schizophrenia.

When thinking about the above-mentioned hypotheses, it raises the question of how the MMNm-like activity is represented in schizophrenic patients compared to normal controls. The purpose of this study was to verify differences in N1m and MMNm-like activity generated in the motor-auditory cross-modal context between schizophrenic patients and normal controls and to compare that activity with the N1m and MMNm in the unimodal context. We hypothesized that MMNm-like activity itself or a comparison with MMNm in schizophrenic patients would present different patterns from normal controls and that it would also have some relevance to the clinical symptoms of schizophrenia, in addition to reproducing the findings on differences in N1m and MMNm between schizophrenic patients and healthy subjects described in previous studies.

## Materials and methods

### Participants

Fifteen patients diagnosed with schizophrenia (SC group) (10 women, 22–41 years old) according to the Diagnostic and Statistical Manual of Mental Disorders, Fifth Edition (DSM-5) and 15 normal control subjects, sex- and age matched, (NC group) (10 women, 22–42 years old) were recruited to participate in this study. All subjects were right-handed and had no history of neurological or audiological disorders. The subjects of the NC group had no evidence of past or present psychiatric disorders (screened with the MINI interview, Japanese version; 29) and no history of schizophrenia or psychosis in a first-degree relative. Table 1 shows the demographic data of both subject groups. Only one patient in the SC group was not administered medications; the other 14 patients were being medicated with antipsychotic drugs (APD) at a mean dose of 529 mg (range 50–1,100 mg) per day of chlorpromazine (CPZ) equivalents (11 patients using atypical APD, 2 using typical APD), and the remaining one patient using 3 drugs including atypical and typical APDs.

**Table 1:** Demographic data of the subjects.

	Control Subjects (*n* = 15)	Schizophrenia Patients (*n* = 15)
Age (years, range; mean ± SD)	22–42; 31.8 ± 7.29	22–41; 32.7 ± 5.86
Gender (male/female)	5 / 10	5 / 10
Educational History (years, range; mean ± SD)	15–16; 15.8 ± 0.43	12–16; 14.2 ± 1.74
CPZ Equivalent Dose of Antipsychotics (mg/day, range; mean ± SD)		0–1,100; 493.7 ± 340.0
PANSS score		
positive (range; mean ± SD)		10–22; 17.5 ± 3.78
negative (range; mean ± SD)		10–22; 19.5 ± 4.24
general (range; mean ± SD)		25–47; 38.8 ± 5.89

SD, standard deviation, CPZ, chlorpromazine, PANSS, Positive and Negative Syndrome Scale.

After the experimental protocol was explained, written informed consent for participation was obtained from all subjects. This experiment was approved by the Ethics Committee of the National Center of Neurology and Psychiatry (Tokyo, Japan), where the study was conducted.

### Procedures

The auditory stimuli consisted of two Shepard tones: I (110 × 2^n^ Hz) and II (155 × 2^n^ Hz). The two tones were synthesized by a sound editing program (Adobe Systems, San Jose, CA, United States). The auditory stimuli of the tones were delivered binaurally to the ears of the subject at a sound pressure level of 80 dB. Our experiment consisted of a motor-auditory (M-A) task and an auditory (A) task (27).

In the M-A task, subjects held a response pad with six buttons, on which only the two target buttons were colored, yellow and blue. They were asked to press either of the two target (yellow or blue) buttons at random with as equal probability (~50%) and interval (~2 s) as possible without counting with the index finger of their left hand. In 85% of the trials, a yellow button press was followed by tone I and a blue button press by tone II. In 15% of the trials (deviants), the I and II tones were reversed for the buttons. The M-A task was preceded by a rehearsal session of a few minutes. In this session, we confirmed that the subjects could execute our instructions and that they had noticed the regularities between their button pressing and the delivered tone.

In the A task, the subjects were asked to listen to tones delivered using a classical oddball paradigm. Standard (tone I, 85%) and deviant (tone II, 15%) tones were presented to the subjects in a pseudorandom order as an oddball paradigm.

During the MEG recording session, the subjects watched a silent movie. In the A task, following the method of measuring MMNm that is often used, the subjects were instructed to divert their attention on the tones. In the M-A task, to control the background activity with A task, the silent movie was presented in the same manner, but now the subjects were instructed to have a reason for the cause-and-effect logic between their choice of button press and the consequent self-generated tones. The presentation of the movie to the subjects was also meant to maintain their alertness, minimize boredom, and reduce eye movements. The screen was placed about 150 cm in front of the eyes of the subjects so that the center of the image was projected slightly below the horizontal line of sight. The response pad for the button pressing was set on the table in front of the subjects so that it would enter their visual field. No subject pushed buttons other than two target buttons in the array of six buttons due to carelessness or mistakes.

### Data acquisition

Auditorily triggered neuromagnetic responses were recorded in a magnetically shielded room using VectorView (Elekta Neuromag, Helsinki, Finland), which has 204 first-order planar gradiometers at 102 measuring sites on a helmet-shaped surface that covers the entire scalp. Auditory stimulus-triggered epochs of 400-ms duration (including a 100-ms pre-stimulus baseline) were filtered online with a band pass of 1–200 Hz and recorded at a sampling rate of 600 Hz. The MEG responses to auditory stimuli, both matched and mismatched with the button presses, were selective averaged together for analysis. The subjects were continuously monitored through two monitoring cameras set up in the shielded room.

The waveforms were filtered offline using a 1- to 40-Hz band pass. The baseline for the waveforms in each MEG channel was defined by a mean amplitude between −100 and 0 ms. Epochs with artifacts exceeding 3 pT/cm in any MEG channel were discarded. Signal space separation (SSS) correction, head movement compensation, and bad channel correction were applied using MaxFilter software (Elekta Neuromag). In addition, epochs were manually inspected and epochs with artifacts were rejected. Table 2 shows the number of stimuli that was finally used in the analysis. There was no significant difference in the number of stimuli in the various conditions of the two groups.

**Table 2:** Number of stimuli.

	Control Subjects (*n* = 15)	Schizophrenia Patients (*n* = 15)
Motor-auditory condition		
Frequent(range; mean ± SD)	347–489; 417.4 ± 53.7	348–623; 433.5 ± 78.2
Rare(range; mean ± SD)	74–104; 87.7 ± 10.6	71–131; 92.3 ± 16.1
Auditory condition		
Frequent(range; mean ± SD)	407–510; 443.4 ± 34.1	422–481; 449.7 ± 29.2
Rare(range; mean ± SD)	74–90; 78.9 ± 5.75	74–87; 78.7 ± 4.62

SD, standard deviation.

### Data analysis

The peak latency of the main component (N1m to standard tones in the A task) was determined for each hemisphere by the time point at which the root mean square of the predefined left and right perisylvian channels reached the maximum between 70 and 140 ms after the onset of the auditory stimulus. The selected channel areas were presented in a prior study (30, 31). The source-strength waveforms in each hemisphere were calculated using the equivalent current dipoles of the main component in each subject. The responses to the auditory stimuli were selectively averaged for analysis. In the M-A task, the responses, both matched and mismatched with the button presses, were selectively averaged together for analysis, and were weighted according to the number of data acquisition. We looked into the delay between the timing of the button press and the auditory sound and found it to be 16 ms. We corrected for the delay before the data analysis.

The N1m responses were measured between 70 and 140 ms after the tone onset. To compare the attenuation effect of the N1m response by button pressing of the NC and SC groups, the responses were calculated by subtracting the value obtained for the standard tones in the M-A task from the value obtained for the standard tones in the A task (following Model 1). Prior to the statistical analysis, we confirmed the attenuation effect of the N1m response from the obtained data of each subject by visual inspection (following Model 0).

The MMNm response was measured between 100 and 250 ms after the tone onset. The latency of MMNm in our analysis was determined based on several reports (16, 18, 20, 32). The MMNm responses were calculated by subtracting the value obtained for standard tones from the deviant tones in the M-A and A tasks, respectively (following Model 2).

The following two types of mixed effect models were applied. Model 0: the maximum value of the N1m to standard tones in the range of latency [70,140(msec)] was included as the outcome, and the group (NC group, SC group), frequency (standard, deviant), left–right (left, right), condition (A task, M-A task), and interactions of these factors were included as fixed effects, and the subject identification data was specified as a random effect. Model 1: the maximum value of the difference of N1m to standard tones in the A task and the M-A task conditions in the range of latency [70,140(msec)] was included as the outcome, and the group (NC group, SC group), frequency (standard, deviant), left–right (left, right), and interactions of these factors were included as fixed effects, and the subject identification data was specified as a random effect. Model 2: the maximum value of MMNm (−like activity) between the standard and deviant conditions in the range of latencies [100,250(msec)] was included as the outcome, and the group (NC group, SC group), condition (A task and M-A task), left–right (left, right), and interactions of these factors were included as the fixed effects, and subject id was specified as a random effect. For the models, the inferences on least square means and their differences for each level of fixed factors were calculated. Significant levels for statistical tests were set as 0.05 and confidence levels were set as 0.95.

In order to explore the relationships between MEG data and the clinical manifestation of schizophrenia, we also calculated Pearson’s correlation coefficients between MEG measures and several clinical variables in the SC group. These included the PANSS scores (positive, negative and general psychopathology score) and APD dosage, which were translated into CPZ equivalent dosage levels. The MEG measures included the N1m suppression rate, MMNm, and MMNm-like activity. The N1m suppression rate was calculated by dividing N1m to standard tones in the M-A task conditions by that in the A task conditions. The values of N1m, MMNm, and MMNm-like activity were used with the peak amplitude of each component averaged over the left and right hemispheres. Significant levels for statistical tests were set as 0.05.

All statistical analyses were conducted with R software ver. 4.1 (R Core Team, Vienna) and SAS ver. 9.4 (SAS Institute Inc., Cary, NC).

## Results

### N1m-attenuation

**Figure 1:**
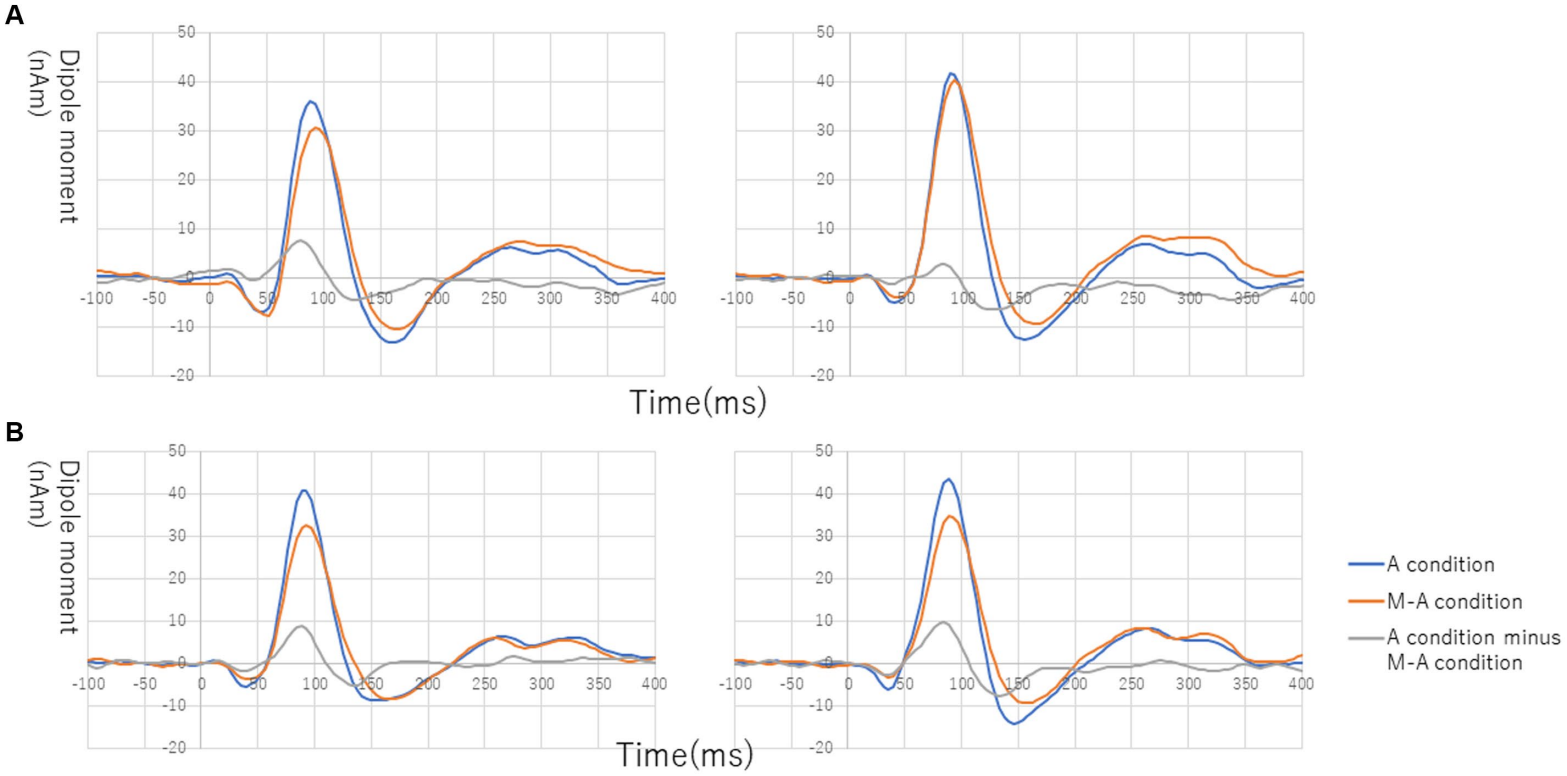
Grand average of source-strength waveforms for the N1m responses to the standard tones: **(A)** normal in the left hemisphere (left) and right hemisphere (right) and **(B)** schizophrenia in the left hemisphere (left) and right hemisphere (right). The blue and red curves plot the A (auditory)-condition and the M-A (motor-auditory) condition, respectively. The gray curve plots the difference between the A condition and the M-A condition.

**Figure 2:**
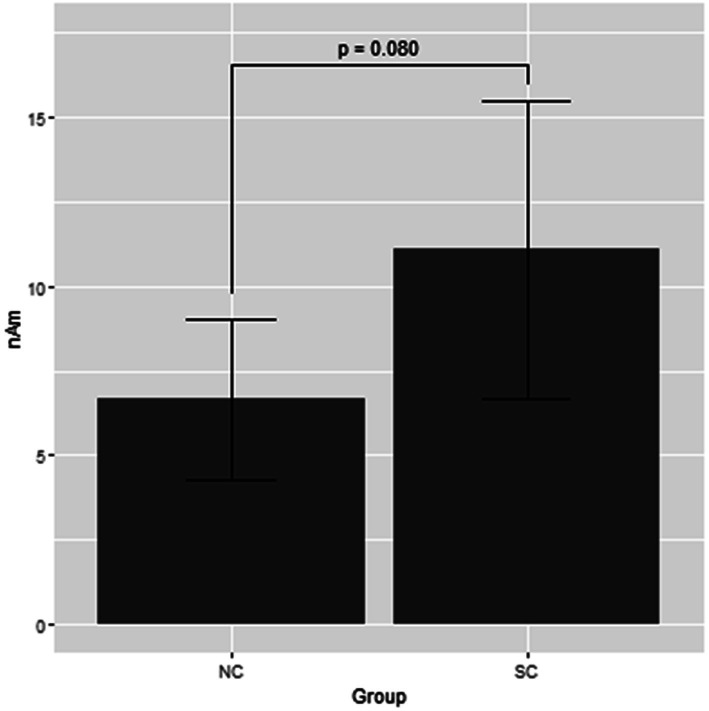
Least square means of attenuation effect of the N1m response to the standard tones with the mixed effect model 1. NC, normal; SC, schizophrenia.

Figure 1 shows the grand average of the distraction of N1m to the standard tones for the M-A and A task in both the NC and SC groups. By visual inspection of the N1m peak responses in NC group, 10 subjects showed the N1m-attenuation effect, 2 showed the opposite effect, i.e., the value of the standard tones in the M-A task was greater than that in the A task, and the remaining 3 showed no clear attenuation effect. In the SC group, 10 subjects showed the N1m-attenuation effect, 1 showed the opposite effect, and the remaining 4 showed no clear attenuation effect. The Model 0 mixed effect model showed that there was no statistically significant difference in the N1m of the standard tones in the M-A task and the A task in the NC group [−3.06 (−6.71, 0.59), *p* = 0.097, where (.) is 95% confidence interval], and that N1m in the M-A task was significantly smaller than that A in the task in SC group [−8.01 (−11.7, −4.42), *p* < 0.001]. The Model 1 mixed effect model showed that there was no statistically significant difference in the N1m-attenuation effects of the NC and SC groups but there was a significant trend towards smaller N1m-attenuation effects in the NC group [−4.43 (−9.44, −0.57), *p* = 0.080], which was the primary analysis target in our study (Figure 2).

### MMNm

**Figure 3:**
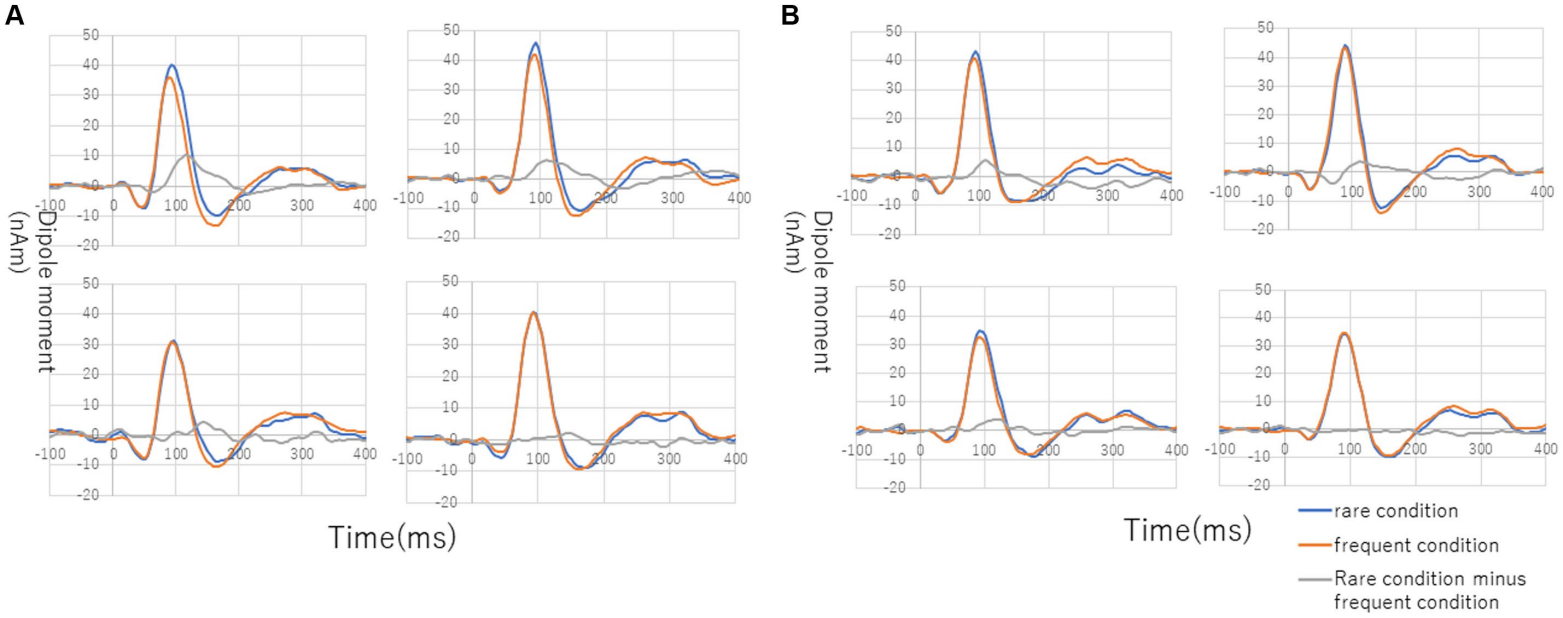
Grand average of source-strength waveforms for the MMNm responses: **(A)** normal in A (auditory) condition (left hemisphere, top left; right hemisphere, top right) and M-A (motor-auditory) condition (left hemisphere, bottom left; right hemisphere, bottom right) and **(B)** schizophrenia in A condition (left hemisphere, top left; right hemisphere, top right) and M-A condition (left hemisphere, bottom left; right hemisphere, bottom right). The blue and red curves indicate rare and frequent conditions, respectively. The gray curves indicate subtraction of frequent from rare conditions.

**Figure 4:**
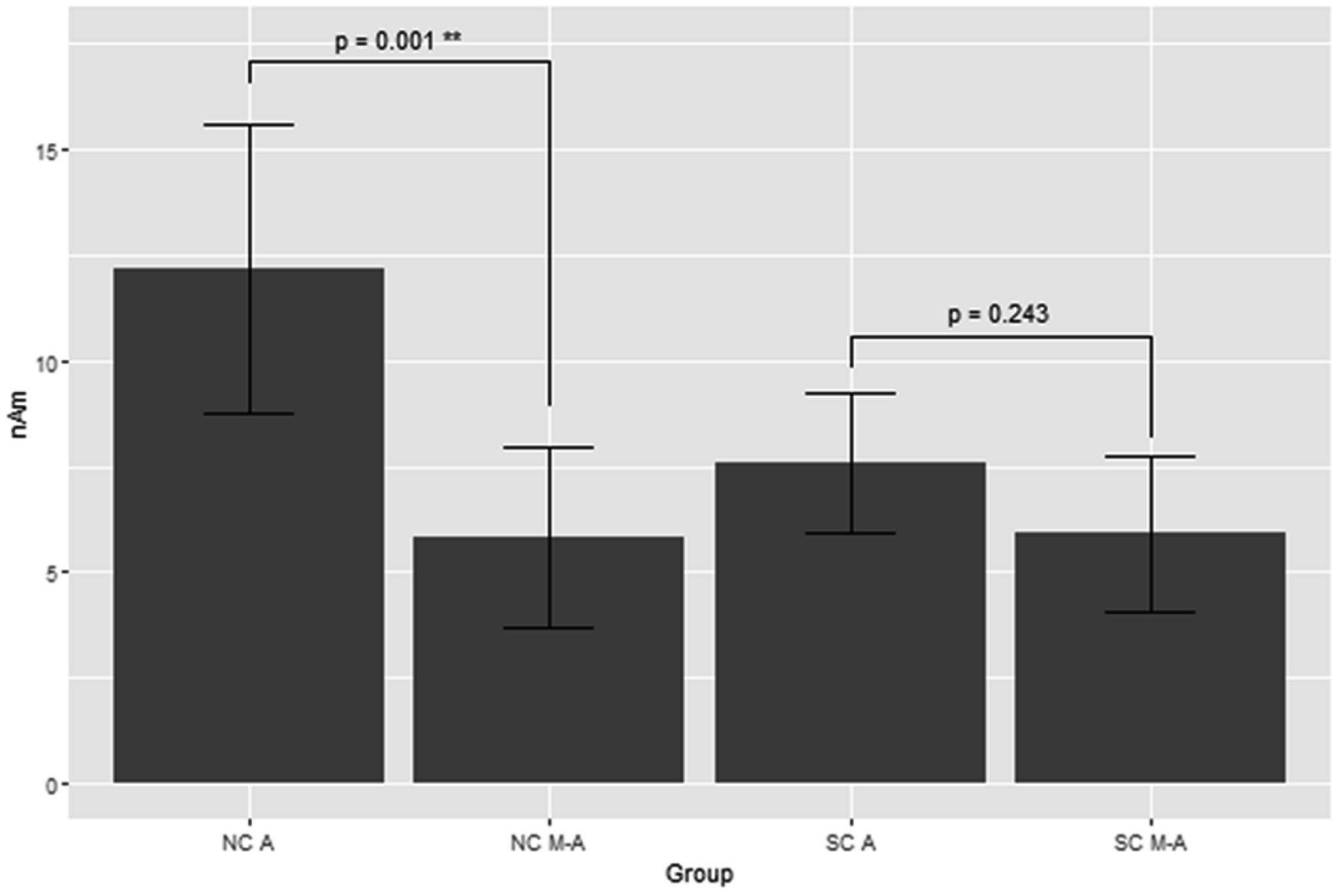
Least square means of the MMNm response with mixed effect model 2. NC A: A (auditory) condition in the normal control, NC M-A: M-A (motor-auditory) condition in the normal control, SC A: A condition in schizophrenia, SC M-A: M-A condition in schizophrenia.

Figure 3 shows the grand average of MMNm (−like activity) (i.e., distraction of deviant minus standard tone) in the M-A and A task in both the NC and SC groups. The Model 2 mixed effect model shows that MMNm in the A task of the NC group was significantly larger than that in the other conditions (M-A task in the NC group [6.35 (2.93, 9.76), *p* = 0.001], M-A [6.27 (2.39, 10.16), *p* = 0.003] and A tasks in the SC group [4.58 (0.77, 8.38), *p* = 0.020]). There was no significant difference in the MMNm (−like activity) of the M-A and A task in the SC group. There was also no significant difference in the MMNm-like activity of the M-A task in the NC group and the various components in the SC group (*p* > 0.05) (Figure 4).

Overall, MMNm in the NC group was significantly larger than the other three components (MMNm-like activity in the NC group, MMNm-like activity in the SC group, and MMNm in the SC group), and there was no significant difference among the remaining three components.

### Correlation

**Figure 5:**
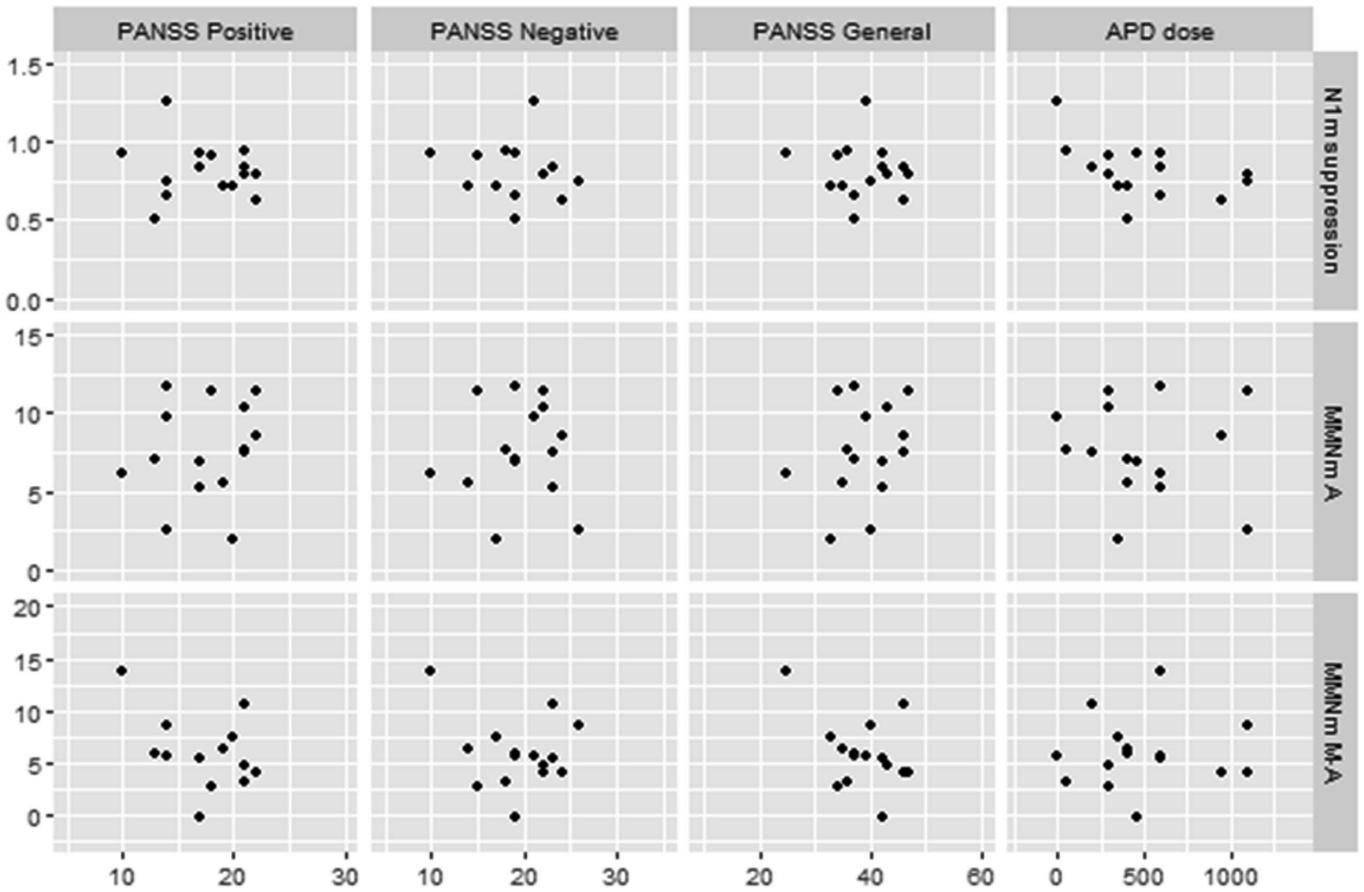
Scatterplots for correlations between MEG data and clinical data in schizophrenic patients. N1m suppression, N1m suppression score; MMN A, mismatch negativity in A (auditory) condition; MMN MA, mismatch negativity in M-A (motor-auditory) condition; PANSS, Positive and Negative Syndrome Scale; APD, antipsychotic drug. APD dose is converted to chlorpromazine equivalents.

The PANSS positive symptoms and general psychopathology scores were moderately negatively correlated with amplitudes of MMNm-like activity (i.e., as scores increase, amplitudes decrease) (*r* = −0.41 and − 0.40, respectively), and the APD dosage was also moderately negatively correlated with N1m suppression rate (i.e., as doses increase, the suppression rate decrease) (*r* = −0.46). However, none of these were statistically significant (*p* > 0.05) (Figure 5).

## Discussion

The aim of this study was to compare N1m-attenuation and MMNm responses to self-initiated tones in a motor-auditory cross-modal context (i.e., the M-A task) with externally initiated tones in a classical oddball context (i.e., the A task) in NC (normal control) and SC (schizophrenic patient) groups.

The main MEG findings showed that (i) there was no significant difference in N1m-attenuation effect for the NC and SC groups, (ii) MMNm in the A task with the SC group was significantly smaller than that in the NC group, and the MMNm(−like activity) in the M-A task was smaller than that in the A task in the NC group, but there were no significant differences in the SC group.

First, we verified the attenuation effect of N1m around the peak latency (70–140 msec) to self-initiated tones in the NC and SC groups. We found that the N1m response was attenuated to self-initiated tones by the button pressing when compared to externally generated tones in both groups. However, some subjects deviated from this rule. This result is in line with that of previous studies (11, 12, 33) but does not fully reproduce them.

Martikainen et al. (12) found that the auditory N1m response was smaller with self-initiated sounds than with externally triggered sounds. This was explained as the existence of a motor-to-sensory forward model (1); and they suggested that this method could provide an objective test for schizophrenic patients, in which dysfunction of the forward model could explain the generation of positive symptoms (1, 2). Ford et al. (7) suggested that reduced N1 suppression in schizophrenic patients reflects a deficit of corollary discharge action. In our study here, there was no significant difference in the N1m attenuation effect of the NC and SC groups, and the trend was rather towards the N1m attenuation effect as greater in the SC group. Therefore, our results are not consistent with their study. It is possible that the reduced auditory N1(m) component in the M-A task may be elicited under the conditions of the forward model, but we could not determine whether the dysfunction of the forward model in schizophrenic patients is present under this N1(m) suppression mechanism. There were some methodological differences between the study of Ford et al. (7) and our current study. That study measured using EEG and presented pure tones (1,000 Hz), while our study measured with MEG and presented Shepard tones and included two tones in the button-pressing task. Most importantly, our study also required a longer experimental time than the Ford et al. study to obtain MMNm in parallel. It may be difficult to obtain robust results that N1(m) attenuation is reduced in schizophrenic patients in the simple shared setting of comparing sounds delivered by button pressing and passive listening. To obtain reproducible results, it may be more reasonable to set up more methodologically rigorous rules or to adopt methods with more research, such as the “Talk/Listen” paradigm (34–36).

Second, we found that the MMNm was smaller in the schizophrenic patients than in the normal control subjects in the single oddball paradigm, in line with many previous studies. On the basis of the assumption of “better-known facts” (17, 37–40), we further tested whether there was a difference in the MMNm component in the M-A and A tasks between the NC and SC groups.

Auditory MMN(m) is elicited by infrequent (deviant) sounds occurring in a sequence of repetitive frequent (standard) sounds. It is believed that MMN(m) represents a neural process of mismatch detection between the deviant auditory input and a sensory memory trace developed by the standard stimuli (32, 41). Most studies have investigated MMN(m) in the auditory unimodal oddball paradigm, and MMN(m) has usually been studied as an automatic pre-attentive response under the passive listening conditions specified by an external context. However, some studies have reported that infrequent audiovisual incongruence also elicits “MMN(m)-like” activity (30, 31, 42). These results suggest that auditory expectant imagery from visual cues elicited MMNm-like activities when expectations were violated. Yumoto et al. (27) verified whether deviant occurrences in a motor-auditory cross-modal context could also mediate such prediction-driven MMNm-like activities. They reported that the deviant tones generated by arbitrary self-initiated button pressing also elicited MMNm-like activity, and suggested that the MMNm-like activity may represent a detection process for prediction errors relevant to internal model reformation.

In our present study, we found MMNm in the A (i.e., auditory listening) task and the MMNm-like activity in the M-A (i.e., motor-auditory cross-modal) task in both the NC and SC groups. In the NC group, the MMNm-like activity in the M-A task was significantly lower than MMNm in the A task. However, in the SC group, there was no significant difference between the MMNm-like activity in the M-A task and the MMNm in the A task. The MMNm-like activity in the M-A task in the SC group was smaller than the MMNm in the A task in the NC group, but there was no significant difference between the MMNm-like activity in the M-A task in the SC group and that in the M-A task in the NC group.

It is difficult to explain the reasons for the reduction in the MMNm-like activity compared to the MMNm in the NC group because there are few findings about this response to deviant occurrences in the motor-auditory cross-modal context even in normal subjects. One explanation could be that it is simply due to subject stimulus discrimination accuracy in the tasks. It is considered that it may be easier to detect deviant events and to match the previous sensory memory in the context of the unimodal oddball paradigm than the cross-modal context, and high-demand tasks could reduce the MMNm amplitude. However, Bendixen and Schröger (43) emphasized that the auditory system has the ability to extract and apply abstract rules in a fast and efficient manner and showed that on these grounds, changes in MMN were minimally affected, even on a complex paradigm assuming that deviant stimuli change over time. They also suggested that there is a dissociation between automatic detection (MMN data) and conscious detection (behavioral data), but it is not significant. Therefore, stimulus complexity may not be a valid explanation why the MMNm was attenuated in the NC group in our study. A further potential explanation could be that MMNm mainly operates as an automatically pre-conscious relevance filtering mechanism, and that it is more compatible with external stimuli regardless of the intentions of the responding subject, as many studies have shown. It is possible that the mismatch detection is also part of a forward model to discriminate self-generated deviance from an externally generated deviance. It is believed that MMN(m) involves a system for the detection of information crucial to survival, such as alerting to potential threats in the environment (28, 44). If the essential mechanism of MMN(m) is like this, the result of our study that the mismatch reaction generated from an external deviation is greater than that of a self-generated internal deviation in normal subjects could offer a possible explanation. We may infer that MMN(m) also plays some role in distinguishing between internal and external events (in other words, prevent excessive linkage between self-action and external events) under the forward model in normal subjects.

The sensory consequences of self-generated movements, such as a tone delivery following the pressing of a button, have been regarded as a model of internally generated experiences, with externally generated sensory inputs regarded as externally caused passive experiences (1, 2, 7, 34). Several studies have shown that the response to self-produced stimulation was attenuated compared to that of externally generated stimulation and that the degree of attenuation was smaller in schizophrenic patients than in normal subjects. These results were often discussed in relation to misattribution of inner experiences to external agents in schizophrenic patients. Synofzik et al. (45) suggested that compared to normal persons, schizophrenic patients tend to rely more strongly on external cues to realize predictions and that they may over-attribute external events to their own agency. Voss et al. (22) suggested that the predictive structuring link in schizophrenic patients is impaired, and therefore, that they show an over-strong linkage between internally generated actions and external sensory events. The results of Voss et al. (22) could support a finding that schizophrenic patients rely on feedback rather than on the forward model for their perceptions and taken together these studies may show that schizophrenic patients have difficulty in maintaining neutrality to external input. Possibly, schizophrenic patients display a dysfunction of the mechanism that clearly recognizes an external event as such (i.e., something that is “external, and not internal”), as would be the case with normal subjects. If this mismatch response difference also plays a role in the discrimination between self-generated events and external events in normal subjects, the response pattern in schizophrenic patients could disrupt a precise discrimination between self and external experiences.

Our results would be in line with those of previous studies which suggest that the generation of prediction errors remain unchanged in most schizophrenic patients (20) and that they tend to depend on the estimates of prediction errors to deal with feedback from the outside (45). In other words, compared with the situation in normal subjects, the detection of prediction errors in schizophrenic patients tends to be influenced by internal self-generated rules derived from external cues, and that prediction errors may be uncertain in the passive automatically “echoic” conditions such as the methods used in traditional MMNm research. The excessive linkage between self-action and external events that causes a failure of the self-monitoring in schizophrenic patients could be attributed to a dysfunction of the forward model mechanisms in prediction errors rather than dysfunction of the prediction error itself. This may have led to the result in this study. At least, it cannot be concluded that the incomplete generation of MMN(m) in schizophrenic patients simply reflects a disability of their auditory change detection.

Here, the question arises as to the implications of behavioral differences of MMNm (−like activity) between normal subjects and the schizophrenic patients in our study. We found the attenuation of MMNm associated with the motion-related forward model paradigm in normal subjects. The fact that the attenuation of MMNm is decreased in schizophrenic patients is noteworthy. Recently, some studies suggest that the decreased MMN(m) in schizophrenic patients reflects their altered predictive coding (46–49) in which the brain is constantly trying to minimize the discrepancy between actual sensory input and internal representations of the environment (28).Our result may reflect that schizophrenic patients perform predictive coding differently from healthy controls after processing (or failing to process) sensory prediction errors in external factors and self-generated stimuli. It remains unclear why changes in MMN(m) responses during unimodal and cross-modal contexts differ in healthy controls and schizophrenic patients. However, there is little knowledge on the properties of MMN(m) in cross-modal contexts. It may be a secondary product of differences in information processing at the N1(m) level between healthy controls and schizophrenic patients, or may be due to dysfunction of MMN(m) itself in schizophrenic patients, or both.

It is possible that the difference in the waveforms of the MMNm-like activity in the M-A task in the present study can be interpreted as the later component of N1m rather than MMNm because the latency mainly, showing a significant difference from 100 to 130 msec (Figure 1, grand average). SanMiguel et al. (13) suggested that effects of N1 suppression due to prediction *via* the forward model were most related to the sensory- non-specific N1 components at the Cz electrode in EEG. In fact, there are competing hypotheses about the neural mechanisms of MMN(m) generation. The most common interpretation is that MMN(m) represents the change detection process involved in a memory-trace effect, which is functionally and spatially distinct from N1(m) generation (50). Another hypothesis is that MMN(m) results from differences in the adaptation of the N1(m) responses to standard and deviant stimuli (51). These conflicting ideas indicate that the relationship between N1(m) and MMN(m) have not been fully clarified although the predictive coding model could provide a common framework for accepting both hypotheses (52, 53). It is unknown whether the two mechanisms can be explained as a series of individual phenomena or as an overlap of mutually independent phenomena.

Additionally, we found several correlations between MEG data and clinical symptoms in schizophrenic patients. First, increases in APD dosage correlated with decreases in the N1m suppression rate. To our knowledge, there are no previous studies that have discussed the relationship between N1(m) suppression and APD doses. It is possible that APD administration may affect neuro-electro-magnetic data by its efficacy (improvement of psychosis) or side effects, such as drowsiness. However, we found no difference in N1m levels between normal controls and schizophrenic patients, as described above. Therefore, it is still unclear to what extent this result is relevant to the pathophysiology of schizophrenic patients. Second, we found that increases of PANSS positive and general psychopathology scores were correlated with decreased MMNm-like activity. However, the correlation did not reach statistical significance. Several previous studies have discussed the association between the PANSS score and MMN. Fisher et al. found that PANSS positive symptom scores were correlated with the duration of the MMN amplitude (24) and intensity MMN latencies (26). They further suggested that schizophrenic patients with auditory hallucinations which is the main symptom among positive symptoms showed significantly smaller MMN than the duration deviants without auditory hallucinations as well as than normal controls (23, 25). Riel et al. (54) suggested that lower PANSS general psychopathology scores were associated with larger MMN amplitudes. However, some reports found no significant association between MMN impairment and the severity of symptoms in schizophrenic patients (55). Although the results of our study cannot be simply compared with previous studies due to different modalities and analytical methods, our results suggest that MMNm-like activity and MMNm are not homologous in their correlation with individual patient psychiatric symptoms. Our results appear to infer that MMNm-like activity may need to be further investigated as an additional option and indicator of schizophrenic pathophysiology. The correlation analysis also mentions pairs that may not have been significant due to the small sample size. Verification of these results is a subject for future research.

Small sample size of subjects reduces the statistical power of the current study. This issue, however, also does not seem to correspond to the diversity of the diseases of schizophrenics. Considering the psychiatric symptom rating scale (PANSS score) and antipsychotic drug dosage regimen, we believe that the schizophrenic patients in our data are representative of outpatients who live in the community despite having some residual psychiatric symptoms. Still, the heterogeneous nature of schizophrenics including epidemiologically, symptomatically, and possibly genetically, will require data from a larger sample to obtain general findings for use as a biomarker. Further research is needed.

In conclusion, we could not find significant differences in the attenuation effect of the N1m to self-initiated sounds in both normal and schizophrenic subjects as discussed in previous studies. Therefore, we could not support the validity of the attenuation effect of the N1m as an indicator of dysfunction of the forward model in schizophrenic patients in this study. The auditory MMNm-like activity evoked by the self-triggered cross-modal events is attenuated compared to the MMNm evoked by the external events in the normal controls. The results could suggest that the auditory mismatch response plays some role in distinguishing between internal and external events. Moreover, we did not find this attenuation effect of MMNm in the schizophrenic patients. Differences in the response patterns of MMNm and MMNm-like activity between healthy controls and schizophrenic patients may reflect differences in processing the predictive coding under forward model generation in both groups. This phenomenon could prompt schizophrenic patients to experience the boundaries of external and internal stimuli differently compared to healthy controls. This may support the hypothesis that schizophrenic patients tend to misattribute their inner experience to external agents, thus leading to their characteristic symptoms.

## Data availability statement

The original contributions presented in the study are included in the article/supplementary material, further inquiries can be directed to the corresponding author.

## Ethics statement

The studies involving humans were approved by National Center of Neurology and Psychiatry. The studies were conducted in accordance with the local legislation and institutional requirements. The participants provided their written informed consent to participate in this study.

## Author contributions

MO: conceptualization, methodology, recruit, software, writing-original draft preparation, writing-reviewing, and editing. MY: conceptualization, methodology, software, supervision. YK: Methodology, software, supervision. KM: statistical analysis. All authors contributed to the article and approved the submitted version.

## Funding

This study is funded by the Intramural Research Grant (24—11) for Neurological and Psychiatric Disorders of National Center of Neurology and Psychiatry.
